# Supplementary material for: Pomegranate Peel Extract Differently Modulates Gene Expression in Gingiva-Derived Mesenchymal Stromal Cells under Physiological and Inflammatory Conditions
Source: Int J Mol Sci. 2023 Oct 21;24(20):15407. doi: 10.3390/ijms242015407 (PMC10607867; doi:10.3390/ijms242015407)
Supplement: Supplementary file 1 [file ijms-24-15407-s001.zip › ijms-2617898-supplementary.pdf]

Supplementary Materials

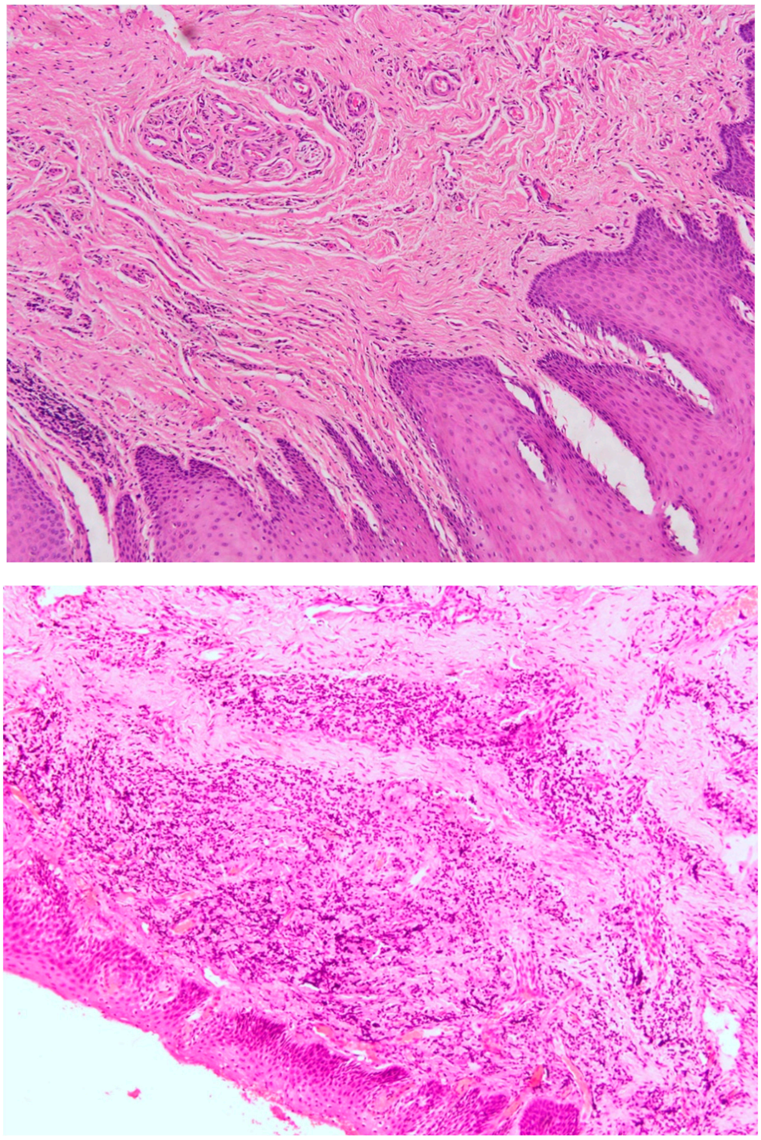

Supplementary Figure S1. Histology of healthy human gingiva (upper) and periodontitis-affected gingiva (bottom). Manifications: x 200

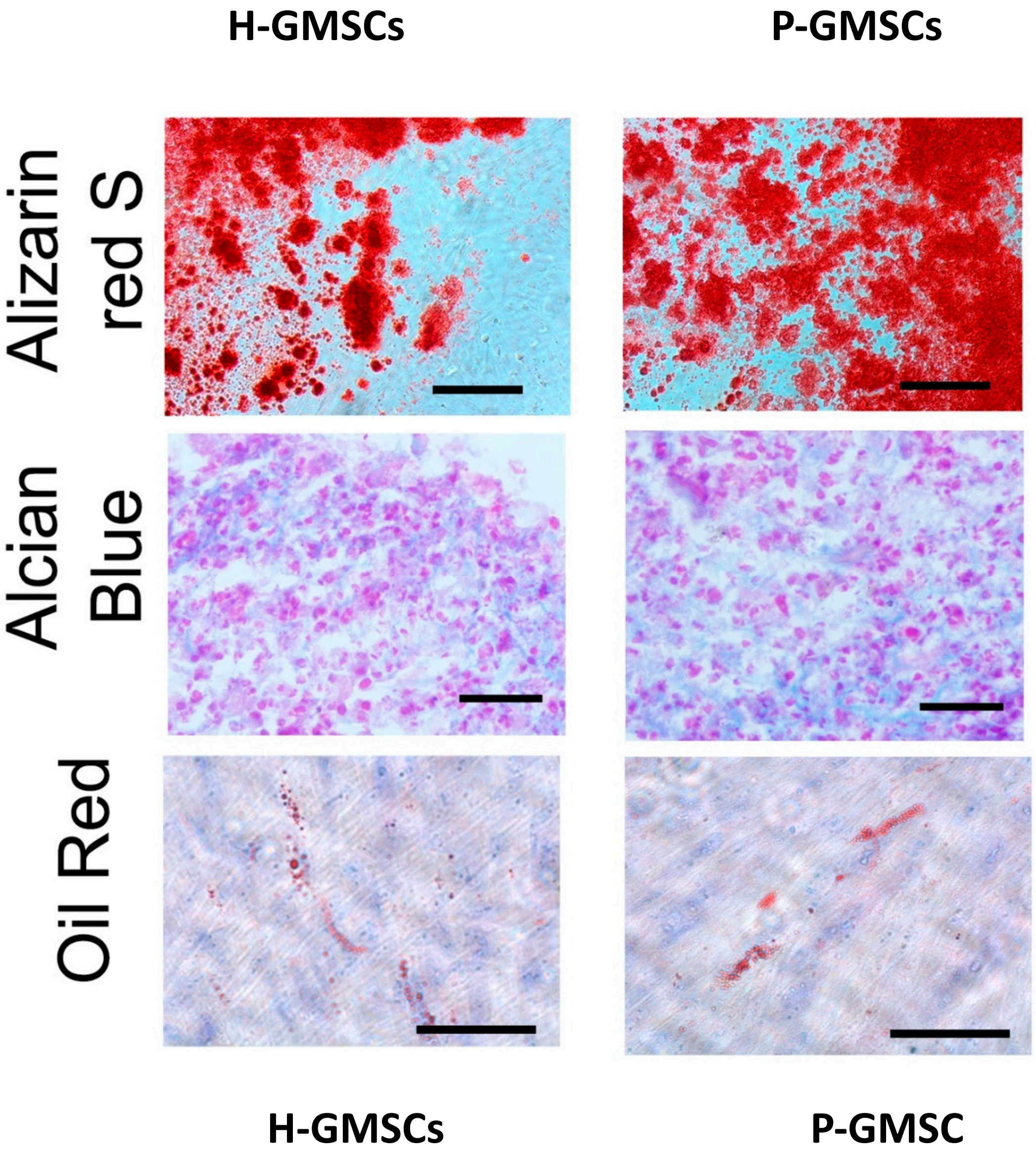

Supplementary Figure S2. Osteogenic, chondroblastogenic and adipogenic differentiation potential of GMSCs. The scale bars for Alizarin red S indicate 100 µm, and for Alcian Blue and Oil Red they indicate 50 µm. Published by: Bekić et al., Mesenchymal Stromal Cells from Healthy and Inflamed Human Gingiva Respond Differently to *Porphyromonas gingivalis*. *Int. J. Mol. Sci.* **2022**, *23*, 3510. https://doi.org/10.3390/ijms23073510. (With permission of MDPI)

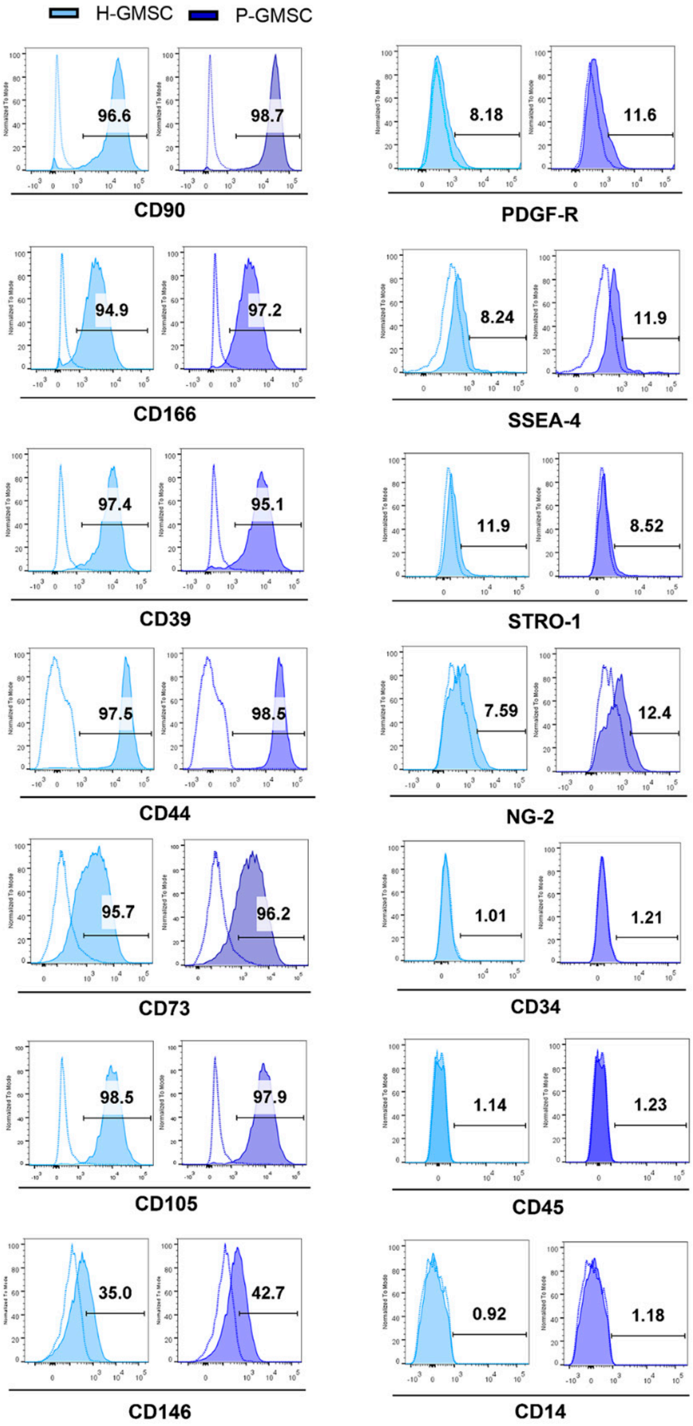

Supplementary Figure S3. Representative histograms of marker expression by GMSC lines. Published by: : Bekić et al., Mesenchymal Stromal Cells from Healthy and Inflamed Human Gingiva Respond Differently to *Porphyromonas gingivalis*. *Int. J. Mol. Sci.* **2022**, *23*, 3510. https://doi.org/10.3390/ permission of MDPI)

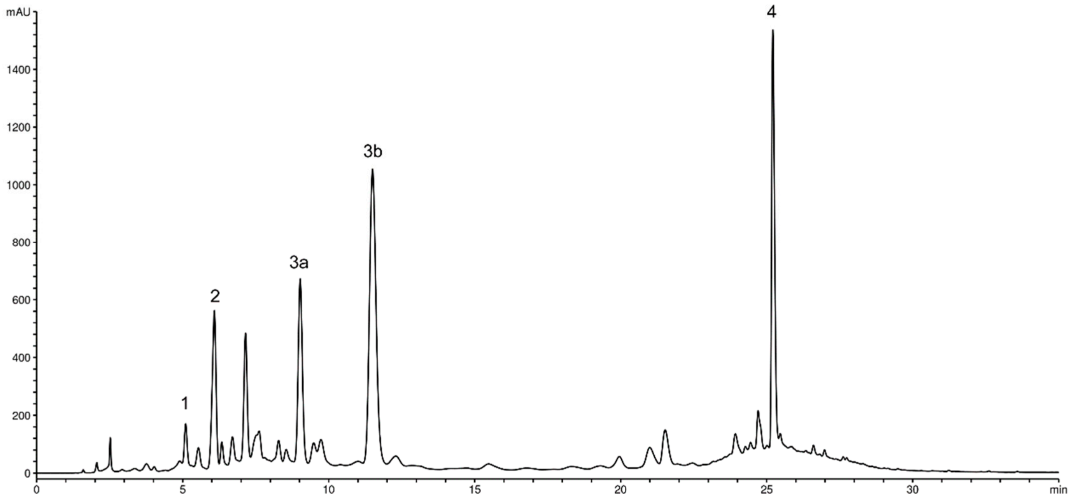

Supplementary Figure S4. HPLC of PoPEx. 1. Gallic acic; 2. Punicalin; 3a. Punicalagin-α; 3b. Punicalagin-β; 4. Ellagic acid. Published by: Čolić et al., Immunomodulatory Properties of Pomegranate Peel Extract in a Model of Human Peripheral Blood Mononuclear Cell Culture. *Pharmaceutics, 14*(6). https://doi.org/10.3390/pharmaceutics14061140. (With permission of MDPI)
